# Long-Term Maternal and Child Outcomes Following Postnatal SSRI Treatment

**DOI:** 10.1001/jamanetworkopen.2023.31270

**Published:** 2023-08-29

**Authors:** Chaoyu Liu, Eivind Ystrom, Tom A. McAdams

**Affiliations:** 1 Social, Genetic and Developmental Psychiatry Centre, Institute of Psychiatry, Psychology and Neuroscience, King’s College, London, England; 2 PROMENTA Research Center, Department of Psychology, University of Oslo, Oslo, Norway; 3 Department Mental Disorders, Norwegian Institute of Public Health, Oslo, Norway; 4 Pharmaco-Epidemiology and Drug Safety Research Group, School of Pharmacy, University of Oslo, Oslo, Norway

## Abstract

**Importance:**

Although selective serotonin reuptake inhibitors (SSRIs) are recommended for postnatal depression treatment, there is a lack of evidence regarding long-term maternal and child outcomes following postnatal SSRI treatment.

**Objective:**

To examine whether postnatal SSRI treatment moderated postnatal depression–associated maternal and child outcomes across early childhood years.

**Design, Setting, and Participants:**

This cohort study used longitudinal data from the Norwegian Mother, Father and Child Cohort Study. Participating women were recruited in weeks 17 to 18 of pregnancy from 1999 to 2008 and were prospectively followed up after childbirth. Data analysis was performed between December 2021 to October 2022.

**Exposure:**

Postnatal depression diagnosis (a binary indicator of eligibility for treatment) was defined as a score of 7 or greater on the 6-item version of the Edinburgh Postnatal Depression Scale. The Hopkins Symptom Checklist was used as a continuous indicator of and postnatal depressive symptomology at postpartum month 6. Postnatal SSRI treatment was identified using self-reported data at postpartum month 6.

**Main Outcomes and Measures:**

Maternal outcomes included self-reported depression symptomology and relationship satisfaction from childbirth to postpartum year 5. Child outcomes included maternal-report internalizing and externalizing problems, attention-deficit/hyperactivity disorder symptoms, and motor and language development at ages 1.5, 3, and 5 years. A propensity score adjustment method was used to control for prenatal factors associated with postnatal SSRI exposure probability.

**Results:**

Among a total of 61 081 mother-child dyads, 8671 (14.2%) (mean [SD] age, 29.93 [4.76] years) met the criteria for postnatal depression diagnosis, 177 (2.0%) (mean [SD] age, 30.20 [5.01] years) of whom received postnatal SSRI treatment. More severe postnatal depression symptomology was associated with a range of adverse maternal and child outcomes. Focusing analyses only on the postnatal depression dyads indicated that postnatal SSRI treatment attenuated negative associations between postnatal depression and maternal relationship satisfaction at postpartum month 6 (moderation β, 0.13; 95% CI, 0.07-0.19), years 1.5 (moderation β, 0.11; 95% CI, 0.05-0.18) and 3 (moderation β, 0.12; 95% CI, 0.04-0.19), and for child ADHD at age 5 years (moderation β, −0.15; 95% CI, −0.24 to −0.05). Postnatal SSRI treatment mitigated the negative associations between postnatal depression and maternal depression, partner relationship satisfaction, child externalizing problems, and attention-deficit/hyperactivity disorder up to 5 years after childbirth.

**Conclusions and Relevance:**

The results of this large prospective cohort study suggest that postnatal SSRI treatment was associated with a reduced risk of postnatal depression–associated maternal mental health problems and child externalizing behaviors across early childhood years. These findings suggest that postnatal SSRI treatment may bring benefits in the long term to women with postnatal depression and their offspring. This study potentially provides valuable information for clinicians and women with postnatal depression to make informed treatment decisions.

## Key Points

**Question:**

How does postnatal selective serotonin reuptake inhibitor (SSRI) treatment moderate postnatal depression–associated maternal and child outcomes?

**Findings:**

In this cohort study of 61 081 mother-child dyads, postnatal SSRI treatment mitigated the negative associations between postnatal depression and later maternal depression, partner relationship satisfaction, and child externalizing problems and attention-deficit/hyperactivity disorder symptoms up to 5 years after childbirth.

**Meaning:**

The results of this cohort study suggest that postnatal SSRI treatment may bring benefits in the long term to women with postnatal depression and their offspring and is associated with a reduced risk of several adverse maternal and child outcomes associated with postnatal depression.

## Teaser

This cohort study examines the association between postnatal selective serotonin reuptake inhibitor treatment and postnatal depression–associated maternal and child outcomes across early childhood years.

## Introduction

Postnatal depression is a common psychiatric disorder that affects 10% to 15% of women during the first year after childbirth.^1^ In addition to the immediate postpartum period, women with postnatal depression are more susceptible to recurrent depressive episodes in subsequent pregnancies and display higher levels of depression during the years following childbirth.^2,3^ Children born to mothers with postnatal depression show increased difficulties in cognitive development and higher levels of behavioral and emotional problems.^4,5^ Postnatal depression is also associated with long-term partner relationship problems^6^ that are a risk factor for perinatal depression and prolonged postnatal depression courses.^7,8,9^ Persistent postnatal depression is associated with further risk to child development, while symptom remittance is associated with normalization of behavioral problems and psychopathology of exposed children.^10,11^ Such findings highlight the importance of effective interventions for postnatal depression, as it may mitigate some of the negative consequences associated with the condition.

The effectiveness of antidepressants in treating postnatal depression has been supported by data from randomized clinical trials.^12^ Selective serotonin reuptake inhibitors (SSRIs) are the preferred choice due to better safety profiles and tolerance compared with other antidepressants, such as tricyclic antidepressants and monoamine oxidase inhibitors.^13^ However, there is insufficient evidence regarding the long-term consequences of postnatal SSRI use for women and their children.^12^ In addition, conflicting evidence on the association between prenatal SSRI exposure and increased risk of neuropsychiatric problems in offspring may raise concerns about potential negative consequences associated with postnatal SSRI exposure.^14,15,16,17,18,19^ This gap in knowledge potentially contributes to a lack of confidence in medicalizing postnatal depression among clinicians and the affected women.^20^ Data from primary care records in the UK have indicated that the prevalence of postnatal depression is 11%, whereas the initiation rate of SSRIs within 6 months postpartum for postnatal depression is around 3%,^21^ suggesting a gap between diagnosis and medication use. Suboptimal treatment is harmful because poorly controlled postnatal depression has been associated with many negative outcomes in mothers and offspring.^22^

The current study examined maternal mental health outcomes alongside child developmental outcomes associated with postnatal depression up to postpartum year 5. We then explored whether postnatal SSRI treatment moderated any observed associations. Some prenatal factors may confound the associations between postnatal depression, postnatal SSRI treatment, and study outcomes. For example, women experiencing social deprivation and a depression diagnosis prenatally are more likely to receive SSRIs for postnatal depression.^21,23^ Therefore, it is important to control for confounding factors that might partially or completely explain associations between postnatal depression and/or postnatal SSRI use and study outcomes to avoid preexisting differences between treated and untreated women leading to incorrect inference.^24,25^

## Methods

### Sample

Data were obtained from the Norwegian Mother, Father and Child Cohort Study (MoBa), a prospective population-based cohort study in Norway. The MoBa study recruited women who attended a routine ultrasonography examination during week 17 to 18 of pregnancy from 1999 to 2008. Women were invited to participate multiple times when they had more than 1 pregnancy. Among 277 702 invitations sent, more than 95 000 women and 114 000 children were enrolled (participation rate of 41%).^26^ Participating families received questionnaires on child development and maternal health conditions across several assessments from gestational week 17 until last follow-up. The MoBa study obtained a license from the Norwegian Data Inspectorate and approval from the Regional Committee for Medical Research Ethics. All participants provided written informed consent before participation. This study was based on version 12 of the quality-assured data release in January 2019.

The current study included women with data on their depression symptoms at gestation week 30 and postpartum month 6, as well as data on self-reported medication use for mental health problems at postpartum month 6. To maximize the study sample, we included all pregnancies and randomly selected 1 child from each twin birth. A flow chart of the study population is shown in the eFigure in Supplement 1. The study followed the Strengthening the Reporting of Observational Studies in Epidemiology (STROBE) reporting guideline for observational studies.

### Measurement

#### Prenatal Factors

Prenatal maternal factors included self-reported current and/or lifetime depression history, educational level, and income at gestational week 17. Maternal educational level was reported on a scale of 1 to 6, with 1 indicating secondary school and 6 higher education (university and above). Income was the yearly gross income of the mothers, including benefits and other allowance, from 1 as no income to 7 as more than 500 000 NOK (49 353.25 USD).

Prenatal alcohol consumption was collected at gestational week 30. We categorized the response to ever use if the women reported alcohol consumption across any of the 3 trimesters during pregnancy. Ever use tobacco was defined as tobacco smoking during the last 3 months of pregnancy as reported at postpartum month 6.

Prenatal maternal depression symptomology was assessed with the 8-item short version of the Hopkins Symptom Checklist (SCL-8)^27^ at gestational week 30. The SCL-8 captures symptoms of depression and anxiety with a high correlation (*r* = 0.94) with the original instrument. The Cronbach α was estimated at 0.88 in MoBa.^28^

#### Postnatal Depression

Postnatal depression diagnosis (a categorical yes/no indicator of eligibility for treatment) was defined based on the scores on the 6-item version Edinburgh Postnatal Depression Scale (EPDS-6)^29^ as measured at postpartum month 6 for each pregnancy. The EPDS-6 used in MoBa has been shown to correlate highly (*r* = 0.96) with the 10-item full version. The Cronbach α for the EPDS-6 was 0.84 in the MoBa sample. The current study used a cutoff threshold 7 or greater to define postnatal depression diagnosis. This threshold has been validated as identifying postnatal depression cases in previous studies.^30,31^

In addition to using the EPDS-6 as a binary indicator of whether women were eligible for treatment for their postnatal depression, we used the SCL-8 as a continuous measure of postnatal depression symptomatology at postpartum month 6. The correlation of the EDPS and the SCL-8 was 0.7 in the current sample.

#### SSRI-Treated Postnatal Depression Dyads and Non–SSRI-Treated Postnatal Depression Dyads

Mothers were asked to report any medications they had taken at postpartum month 6. The reported medication was identified using the Anatomic Therapeutic Classification (ATC) system of the World Health Organization. Because SSRIs are the most prescribed and researched antidepressants for perinatal mental health problems^12^ and are recommended as the first-line treatment by multiple treatment guidelines,^13,32^ the current study focused exclusively on postnatal use of SSRIs. Mothers with postnatal depression who reported treatment with only anxiolytics (ATC class N05B), hypnotics (ATC class N05C), or non-SSRI antidepressants (ATC classes N06AA, N06AF, and N06AX) were excluded from analyses (n = 81). If the mother identified as having postnatal depression reported SSRI (with ATC class N06AB) use, she was classified as having SSRI-treated postnatal depression. If the mother with postnatal depression did not report SSRI use, she was classified as having non–SSRI-treated postnatal depression. Three mutually exclusive groups of mother-child dyads were included in the main analyses: nonpostnatal depression (52 410 [85.8%]), non–SSRI-treated postnatal depression (8494 [13.9%]), and SSRI-treated postnatal depression (n = 177). We also identified mother-child dyads who reported SSRI use but did not meet the threshold for postnatal depression diagnosis (179 [0.3%]). This group was not included in the main analyses because we were unable to determine if they received SSRI for postnatal depression or other reasons. Due to the few participants who reported SSRI use, analyses comparing individual SSRIs were not performed.

### Maternal Outcomes

Maternal outcomes included maternal depression as assessed with SCL-8 at postpartum years 1.5, 3, and 5. Partner relationship satisfaction was assessed via maternal report on the 5-item short version Relationship Satisfaction Scale at postpartum month 6 and years 1.5 and 3. Items were rated on a 6-point scale to reflect relationship satisfaction (eg, “I am very happy with our relationship”) and partner relationship quality (eg, “my partner is generally understanding”). We used standardized sum scores (mean [SD], 0 [1]) for SCL-8 and Relationship Satisfaction Scale at each point in the regression analyses.

### Child Outcomes

Child internalizing and externalizing behaviors in MoBa were measured with selected items from the Child Behavior Checklist (CBCL)^33^ at ages 1.5, 3, and 5 years. Standardized sum scores (mean [SD], 0 [1]) were calculated separately for internalizing and externalizing behaviors at each point and used in the regression analyses.

Motor and language development were measured with the Ages and Stages Questionnaire.^34^ The Norwegian version of the Ages and Stages Questionnaire has shown high agreement (84%) with standardized assessment tests and effective predictive validity for children with developmental delay.^35^ Standardized sum scores (mean [SD], 0 [1]) for motor and language development were calculated for ages 1.5 and 3 years, respectively.

### Statistical Analysis

Analyses were conducted with R (version 4.0.3; R Foundation). We used the false discovery rate approach at less than 5% to adjust for multiple testing.^36^ Data analysis was performed between December 2021 to October 2022.

#### Propensity Score Model Adjusting for Prenatal Confounding Variables

Based on a review of the literature, we selected several prenatal factors measured in MoBa as potentially associated with postnatal depression diagnosis and postnatal SSRI treatment. We then ran univariable and multiple logistic regressions to establish how these factors performed in the sample and use in the propensity score model. The resultant propensity score was used as an adjusting variable for postnatal SSRI exposure probability in the outcome analysis. The propensity score adjustment approach was intended to emulate randomized assignment of postnatal SSRI treatment to prevent bias from imbalanced prenatal factors between treated and untreated women when studying treatment effectiveness.^37,38^ This study’s propensity score model included maternal age, parity, prenatal depression symptoms, depression history, and maternal education and income levels. More details about the use of the propensity score can be found in the eMethods in Supplement 1.

#### Associations Between Postnatal Depression and Maternal and Child Outcomes and the Moderation of SSRI

We used multilevel models with random intercepts (R package lcmm)^39^ at the maternal level to account for nonindependence in the sample (multiple pregnancies nested within mothers). We examined associations between depression symptomatology as measured with SCL-8 at postpartum month 6 and study outcomes. We used SCL-8 because it was repeatedly measured across prenatal and postnatal periods to reflect depression severity and symptom variation. We ran regressions using a doubly robust propensity score adjustment method. The doubly robust adjustment method included the propensity score plus additional potential sources of prenatal confounding and covariates in a multiple regression to maximize the chance of adequately adjusting for confounding while maintaining statistical efficiency.^25^ Covariates in the fully adjusted models were prenatal alcohol and tobacco use for maternal outcomes and child sex, birthweight, and gestational age for child outcomes.

#### Sensitivity Analyses for Moderation of Postnatal SSRI Treatment

We examined postnatal SSRI moderation of study outcomes after controlling for prenatal SSRI exposure in sensitivity analyses. We also performed additional analyses for 179 mother-child dyads who reported SSRI use but did not meet the threshold for postnatal depression diagnosis.

## Results

### Prenatal Factors Associated With Postnatal Depression Diagnosis and Postnatal SSRI Treatment

Among a total of 61 081 mother-child dyads, 8671 (14.2%) (mean [SD] age, 29.93 [4.76] years) met the criteria for postnatal depression diagnosis, 177 (2.0%) (mean [SD] age, 30.20 [5.01] years) of whom received postnatal SSRI treatment. Descriptive statistics for prenatal factors are presented in Table 1 and Table 2. A multivariable logistic regression showed that a lower education level (odds ratio [OR], 0.86; 95%CI, 0.83-0.89), lower income level (OR, 0.89; 95% CI, 0.86-0.92), prenatal tobacco use (OR, 1.13; 95% CI, 1.02-1.26), lifetime history of depression (OR, 2.06; 95% CI, 1.88-2.21), and higher levels of prenatal depression (OR, 2.35; 95% CI, 2.29-2.42) differentiated women with postnatal depression from those without (Table 1). Table 2 shows prenatal factors associated with SSRI treatment in women with postnatal depression, which include a lower parity (OR, 0.74; 95% CI, 0.59-0.92), a lower educational level (OR, 0.84; 95% CI, 0.71-0.99), a higher level of prenatal depression (OR, 1.25; 95% CI, 1.13-1.36), and lifetime history of depression (OR, 6.98; 95% CI, 4.92-9.98). Eighty of 177 women (45%) in the SSRI-treated postnatal depression group received SSRIs during pregnancy compared with 352 of 8494 (4%) in the non–SSRI-treated postnatal depression group (Table 2).

**Table 1.** Prenatal Maternal and Child Characteristics of Non-PND and PND Dyads

Characteristic	Mean (SD)		Non-PND vs PND^a^			
	Non-PND (n = 52 410)	PND (n = 8671)	OR (95% CI)	*P* value	aOR (95% CI)^b^	*P* value
Maternal prenatal characteristics						
Maternal age, y	30.41 (4.31)	29.93 (4.76)	0.98 (0.97-0.98)	<.001	1.00 (1.00-1.01)	.32
Parity	0.74 (0.85)	0.77 (0.87)	1.05 (1.02-1.08)	<.001	1.01 (0.97-1.05)	.42
Maternal education						
Ranking (scale, 1-6)	4.74 (1.16)	4.38 (1.35)	0.75 (0.73-0.77)	<.001	0.86 (0.83-0.89)	<.001
Maternal income						
Ranking (scale, 1-7)	4.23 (1.32)	3.87 (1.36)	0.76 (0.74-0.78)	<.001	0.89 (0.86-0.92)	<.001
Prenatal alcohol exposure						
Ever used in pregnancy, No. (%)	14 417 (27.6)	2473 (28.5)	1.10 (1.05-1.16)	<.001	1.05 (0.99-1.12)	.09
Prenatal tobacco exposure						
Ever used in pregnancy, No. (%)	2506 (4.8)	772 (8.9)	1.99 (1.83-2.16)	<.001	1.13 (1.02-1.26)	.02
Lifetime depression diagnosis, No. (%)	2247 (4.3)	1389 (16.0)	4.26 (3.96-4.57)	<.001	2.06 (1.88-2.21)	<.001
Prenatal maternal depression (SCL-8^c^)	9.58 (2.13)	12.48 (3.98)	2.52 (2.46-2.58)	<.001	2.35 (2.29-2.42)	<.001
Prenatal SSRI use, No. (%)	619 (1.18)	432 (4.98)	NA	NA	NA	NA
Child characteristics						
Child sex (male), No. (%)	26 732 (51)	4460 (51.5)	NA	NA	NA	NA
Birth weight, kg	3.59 (0.55)	3.58 (0.58)				
Gestational age, wk	39.74 (2.32)	39.68 (2.43)				

Abbreviations: aOR, adjusted odds ratio; NA, not applicable; OR, odds ratio; PND, postnatal depression; SCL-8, Hopkins Symptom Checklist.

^a^ PND was defined as scores of greater than 7 on the Edinburgh Postnatal Depression Scale at postpartum month 6.

^b^ Adjusted multiple logistic regression controlling for all the prenatal factors to identify predictors for PND: reference group was non-PND dyads.

^c^ SCL-8 measures maternal depression symptomology across the prenatal and postnatal period.

**Table 2.** Prenatal Maternal and Child Characteristics of Non–SSRI-Treated PND Dyads and SSRI-Treated PND Dyads

Characteristic	Mean (SD)		Nontreated vs treated			
	Nontreated PND^a^ (n = 8494)	Treated PND (n = 177)	OR (95% CI)	*P* value	aOR (95% CI)^b^	*P* value
Maternal prenatal characteristics						
Maternal age	29.94 (4.76)	30.20 (5.01)	1.01 (0.98-1.04)	.52	1.04 (1.00-1.09)	.04
Parity	0.78 (0.87)	0.68 (0.84)	0.88 (0.73-1.05)	.20	0.74 (0.59-0.92)	.01
Maternal education						
Ranking (scale 1-6)	4.39 (1.35)	4.14 (1.42)	0.85 (0.74-0.97)	.03	0.84 (0.71-0.99)	.03
Maternal income						
Ranking (scale 1-7)	3.88 (1.36)	3.71 (1.39)	0.88 (0.76-1.03)	.16	0.98 (0.81-1.19)	.81
Prenatal alcohol exposure						
Ever used in pregnancy, No. (%)	2425 (28.5)	48 (27.1)	0.92 (0.65-1.29)	.65	0.86 (0.60-1.23)	.35
Prenatal tobacco exposure						
Ever used in pregnancy, No. (%)	747 (8.8)	25 (14.1)	1.68 (1.07-2.54)	.04	0.93 (0.53-1.54)	.87
Lifetime depression diagnosis, No. (%)	1284 (15.1)	105 (59.3)	8.19 (6.05-11.15)	<.001	6.98 (4.92-9.98)	<.001
Prenatal maternal depression (SCL-8^c^)	12.42 (3.92)	15.26 (5.43)	1.41 (1.31-1.52)	<.001	1.25 (1.13-1.36)	<.001
Prenatal SSRI use, No. (%)	352 (4.14)	80 (45.2)	NA	NA	NA	NA
Child characteristics						
Child sex (male), No. (%)	4364 (51.4)	96 (54.2)	NA	NA	NA	NA
Birth weight, kg	3.59 (0.57)	3.53 (0.53)				
Gestational age, wk	39.69 (2.43)	39.27 (2.23)				

Abbreviations: aOR, adjusted odds ratio; NA, not applicable; OR, odds ratio; PND, postnatal depression; SCL-8, Hopkins Symptom Checklist.

^a^ PND was defined as scores of greater than 7 on the Edinburgh Postnatal Depression Scale at postpartum month 6.

^c^ SCL-8 measures maternal depression symptomology across the prenatal and postnatal period.

### Association Between Postnatal Depression and Maternal and Child Outcomes

**Figure 1.**
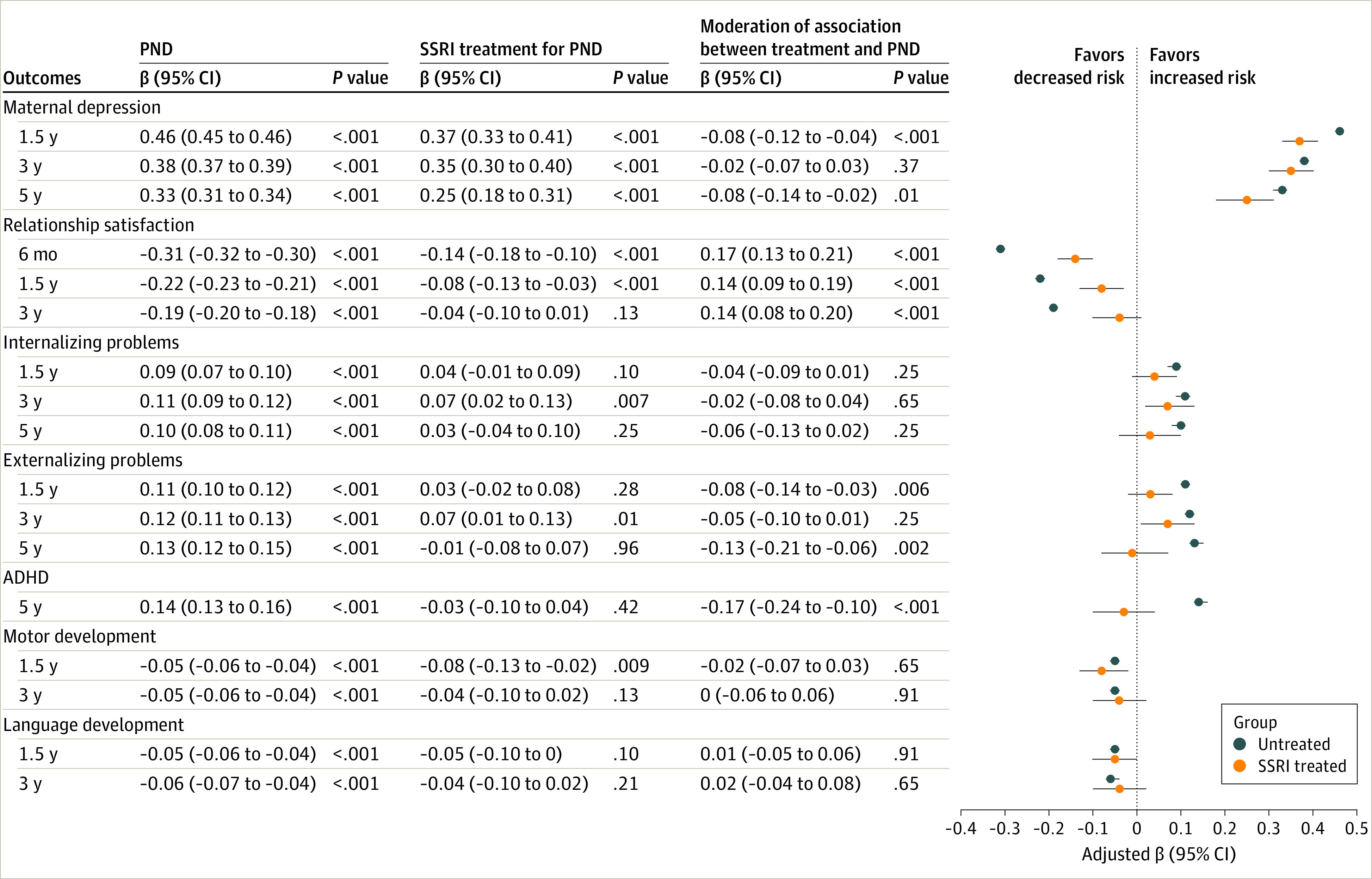
Maternal and Child Outcomes and the Association Between Postnatal Maternal Depression, Selective Serotonin Reuptake Inhibitor (SSRI) Treatment, and the Study Outcomes in the Study Population For postnatal depression (PND), estimated associations with maternal and child outcomes were measured by the Hopkins Symptom Checklist (SCL-8) at postpartum month 6. Mothers postnatally treated with SSRIs (txPND): estimated associations between the SCL-8 and study outcomes in the group of mothers postnatally treated with SSRIs. For moderation, interaction terms were examined between postnatal depression symptoms measured by the SCL-8 and postnatal SSRI use. Estimates presented were the standardized regression coefficient adjusted for prenatal confounding (maternal age, parity, maternal income, prenatal depression and anxiety, lifetime depression, and income and education levels), propensity score, and covariates. Covariates for maternal outcomes included maternal prenatal alcohol and tobacco use. Covariates for child outcomes included child sex, birthweight, and gestational age.

**Figure 2.**
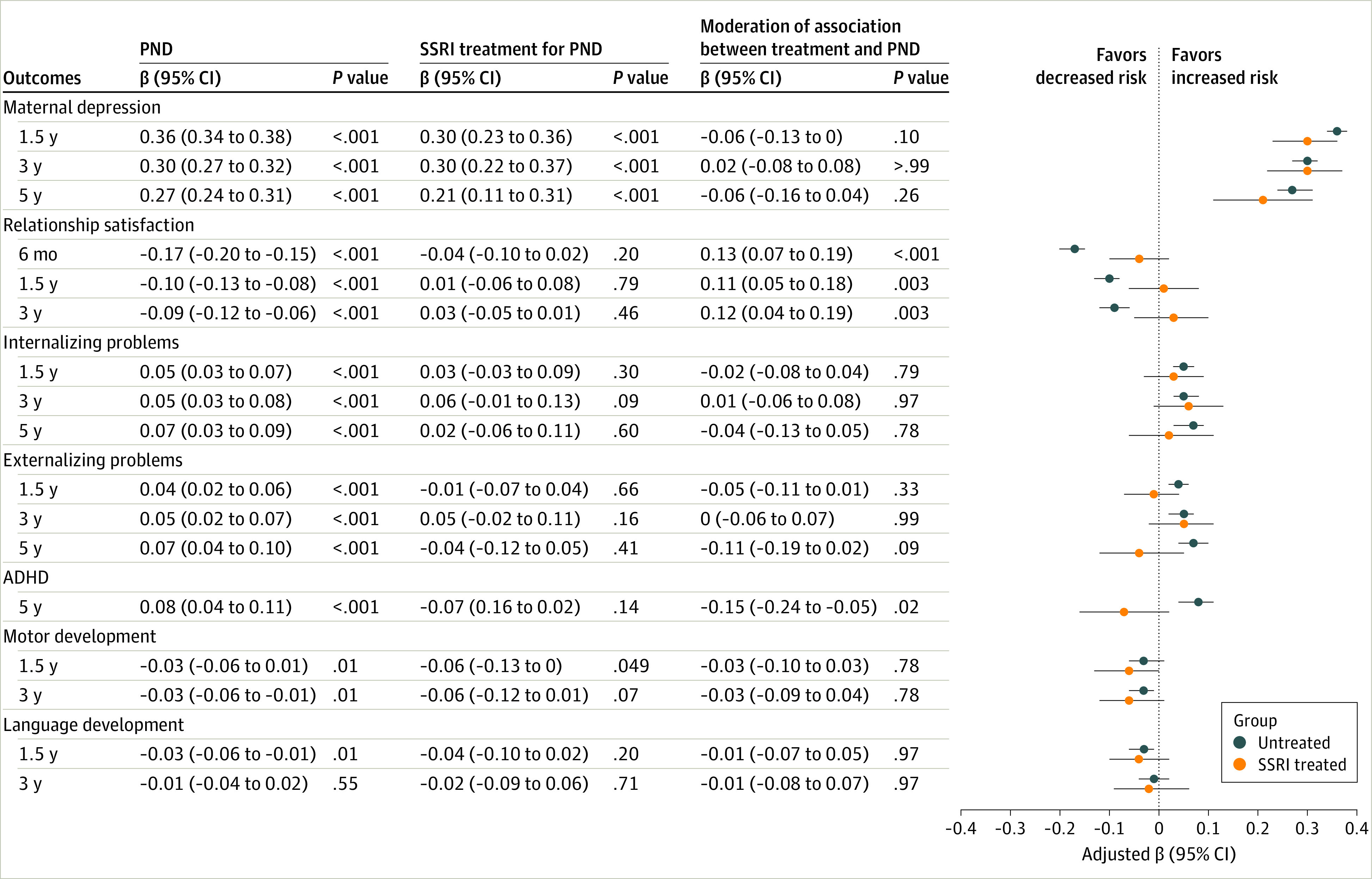
Maternal and Child Outcomes and the Association Between Postnatal Maternal Depression, Selective Serotonin Reuptake Inhibitor (SSRI) Treatment and the Study Outcomes in Mother-Child Dyads Categorized as Eligible to Use SSRIs For postnatal depression (PND), estimated associations with maternal and child outcomes were measured by the Hopkins Symptom Checklist (SCL-8) at postpartum month 6. Mothers postnatally treated with SSRIs (txPND): estimated associations between the SCL-8 and study outcomes in the group of mothers postnatally treated with SSRIs. For moderation, interaction terms were examined between postnatal depression symptoms measured by the SCL-8 and postnatal SSRI use. Estimates presented were the standardized regression coefficient adjusted for prenatal confounding (maternal age, parity, maternal income, prenatal depression and anxiety, lifetime depression, and income and education levels), propensity score, and covariates. Covariates for maternal outcomes included maternal prenatal alcohol and tobacco use. Covariates for child outcomes included child sex, birthweight, and gestational age.

Multiple regression in the whole study population and between postnatal depression subgroups (Figure 1 and Figure 2; eTables 1 and 2 in Supplement 1) showed that postnatal depression severity was associated with higher levels of maternal depression across postpartum years 1.5 to 5 and poorer relationship satisfaction across postpartum month 6 to year 3. Postnatal depression severity was associated with higher levels of child internalizing and externalizing behaviors as measured across ages 1.5 to 5 years, poorer motor and language development at years 1.5 and 3, and attention-deficit/hyperactivity (ADHD) symptoms at age 5 years.

### Postnatal SSRI Treatment Moderation of the Association Between Postnatal Depression and Maternal and Child Outcomes

A moderation analysis in the whole study sample (Figure 1; eTable 1 in Supplement 1) indicated that postnatal SSRI treatment–moderated associations between postnatal depression and maternal depression at postpartum year 1.5 (moderation β, −0.08; 95% CI, −0.12 to −0.04) and postpartum year 5 (moderation β, −0.08; 95% CI, −0.14 to −0.02) and relationship satisfaction at postpartum month 6 (moderation β, 0.17; 95% CI, 0.13-0.21), years 1.5 (moderation β, 0.14; 95% CI, 0.09-0.19) and 3 (moderation β, 0.14; 95% CI, 0.08-0.20). Postnatal SSRI treatment also moderated the associations between postnatal depression and child externalizing behaviors at ages 1.5 (moderation β, −0.08; 95% CI, −0.14 to −0.03) and 5 years (moderation β, −0.13; 95% CI, −0.21 to −0.06) and ADHD at age 5 years (moderation β, −0.17; 95% CI, −0.24 to −0.10).

Focusing analyses only on the postnatal depression dyads (Figure 2; eTable 2 in Supplement 1) indicated that postnatal SSRI treatment attenuated negative associations between postnatal depression and maternal relationship satisfaction at postpartum month 6 (moderation β, 0.13; 95% CI, 0.07-0.19), years 1.5 (moderation β, 0.11; 95% CI, 0.05-0.18) and 3 (moderation β, 0.12; 95% CI, 0.04-0.19), and for child ADHD at age 5 years (moderation β, −0.15; 95% CI, −0.24 to −0.05).

### Sensitivity Analyses for Moderation of Postnatal SSRI Treatment

The associations between postnatal depression and maternal and child outcomes were similar after controlling for prenatal SSRI use. Although some moderation became nonsignificant, confidence intervals before and after adjusting for prenatal SSRI exposure overlapped (eTables 3 and 4 in Supplement 1).

Additional analyses in mothers who reported SSRI use but who did not meet criteria for postnatal depression are shown in eTables 5 and 6 in Supplement 1. Mothers in this group had better mental health outcomes and higher relationship satisfaction throughout the follow-up compared with mothers in postnatal depression subgroups. Children in this group showed fewer externalizing problems but poorer language development at age 1.5 years compared with non–SSRI-treated postnatal depression dyads (eTable 5 in Supplement 1). A moderation analysis showed that postnatal SSRI treatment mitigated the negative association between postnatal depression symptoms and maternal depression at postpartum year 5 even when the level of postnatal depression did not meet the diagnostic threshold (moderation β, −0.35; 95% CI, −0.58 to −0.13) (eTable 6 in Supplement 1).

## Discussion

We used data from a prospective population-based cohort to study whether postnatal SSRI treatment moderated any observed associations between postnatal depression and long-term maternal and child outcomes. Overall, the analyses suggested that postnatal SSRI treatment was associated with a reduced risk of subsequent maternal depression and child externalizing behaviors and ADHD that was associated with postnatal depression. We found no evidence to suggest that postnatal SSRIs conferred an increased risk for childhood psychopathology or motor and language delay in mother-child dyads affected by postnatal depression.

### Differentiation of Postnatal Depression and the Postnatal Use of SSRIs With Prenatal Factors

This study and previous literature suggest that depression and anxiety during pregnancy and a previous history of depression conferred the largest risks of postnatal depression, greater than factors such as pregnancy-related complications and socioeconomic adversity.^40^ The strong risk (2 times increased) associated with a lifetime depression diagnosis was observed independently of the levels of prenatal depression in the current sample. Such findings suggest that prenatal screening for history of depressive disorder can be useful in signposting a heightened risk of postnatal depression. Lifetime history of depression was also independently associated with the likelihood of postnatal SSRI use in women with postnatal depression. In addition, similar to previous studies, we found that lower parity and lower maternal education levels were associated with postnatal SSRI use.^41,42^ This suggests that the use of SSRI for postnatal depression also depends on social and personal factors, such as awareness of mental health problems and the availability of other nonpharmacological intervention.^21,43^

### Postnatal Depression and Maternal and Child Outcomes

Higher postnatal depression symptomatology was associated with elevated levels of maternal depression and relationship dissatisfaction up to 5 years after childbirth in this study’s sample. Partner relationship dissatisfaction is an established risk factor for the onset and persistence of postnatal depression.^7,44^ This 2-way association between partner relationship dissatisfaction and maternal depression warrants further clinical attention.^45^ The current study and others have identified associations between postnatal depression and impairment of cognitive, emotional, and behavioral functioning in exposed children across childhood.^11,46,47^ These findings highlight the importance of early intervention for postnatal depression to potentially prevent unfavorable outcomes in offspring in the long term.^3,10^

### Moderation of SSRI Treatment on the Association Between Postnatal Depression and Postnatal Outcomes

Adequate treatment for postnatal depression is important given its negative associations across many outcomes in mothers, children, and the wider family. However, limited evidence on the long-term consequences associated with postnatal SSRI use in offspring may be associated with treatment hesitancy.^12,20,23^ The prevalence of postnatal SSRI use among women with postnatal depression in this study’s sample was 2% (177 of 8617). Although we were unable to determine how many women received nonpharmacological treatment, the low prevalence of use of SSRIs among women with postnatal depression suggests possible inadequate treatment of the condition. The current study provided evidence on long-term consequences associated with the use of SSRI for postnatal depression. We found that postnatal SSRI treatment mitigated the negative associations between postnatal depression and offspring ADHD symptoms in children born to mothers with more pronounced postnatal depression symptoms. The benefits associated with postnatal SSRI treatment were also found after controlling for prenatal SSRI exposure and in mother-child dyads who did not meet postnatal depression diagnosis criteria. These findings support the notion that adequate treatment of postnatal depression may predict normalization in child behavioral problems associated with the condition.^48^ Additionally, we found no evidence that SSRIs were associated with an increased risk of delayed motor and language development associated with postnatal depression up to age 5 years.

### Limitations

A strength of this study was the prospective design with repeated measurement in a large mother-child cohort. The findings potentially fill the gap in the literature in providing data on long-term child outcomes associated with postnatal SSRI use among women with postnatal depression. Notwithstanding the strengths, several limitations warrant mention. First, the study used maternal self-reported data from a population-based cohort. Previous research indicates that mothers with depression tend to overreport emotional and behavioral problems of their children and the reporting errors tend to increase with the severity of depression.^49,50^ Women treated with SSRIs in our sample presented with more severe depression throughout the follow-up period; therefore, they may have systematically overreported problematic symptoms in their children. However, it also suggests that the positive moderation associated with SSRIs are less likely to be associated with depression-related reporting bias. Future studies should incorporate different sources of reports to evaluate this issue. Second, the current study used self-reported medication history for SSRI use. Although self-reported medication use may be potentially inaccurate, the concordance between self-reports and dispense records from the national prescription database for perinatal SSRI use is high in the MoBa sample.^51^ Third, the dosage of SSRI and information on breastfeeding were not available in the current study. Although SSRIs prescribed as the first-line treatment for postnatal depression, such as sertraline, have been shown to have very low or undetectable infant plasma concentration,^52^ to better understand effects associated with postnatal SSRIs, it would be preferable to account for infant exposure in analyses. Another limitation regarding data availability was that mothers did not report whether they received nonpharmacological interventions alongside (or instead of) SSRIs. The outcomes associated with SSRIs may have been exaggerated if the women simultaneously received other forms of treatment for postnatal depression or weakened if women in the non–SSRI-treated dyad presented with better outcomes due to other interventions. We acknowledge the aforementioned limitations pertinent to observational study design, and our findings should be interpreted cautiously. However, given the costs and ethical concerns that would be associated with conducting large-scale randomized clinical trials for the long-term consequences associated with postnatal SSRI use, we suggest that our findings represent a step forward in informing care and management for postnatal depression.

## Conclusions

In this longitudinal cohort study we aimed to increase understanding of the long-term outcomes associated with postnatal SSRI treatment of mother-child dyads affected by postnatal depression. Postnatal depression was associated with poorer maternal and child outcomes up to postpartum year 5. However, associations between postnatal depression and some unfavorable outcomes were attenuated by SSRI treatment. While our findings need to be replicated, we believe that they add important information regarding long-term outcomes associated with SSRI use for postnatal maternal depression.

## Notes

Supplement 1.
eMethods.
**eTable 1. **Maternal and child outcomes and the association between postnatal maternal depression, SSRI treatment and the study outcomes in the whole study population **eTable 2. **Maternal and child outcomes and the association between postnatal maternal depression, SSRI treatment and the study outcomes among PND dyads **eTable 3. **Association between postnatal maternal depression, SSRI treatment and the study outcomes in the whole study sample controlling for prenatal SSRI use **eTable 4. **Association between postnatal maternal depression, SSRI treatment and the study outcomes in the postnatal depression sample controlling for prenatal SSRI use **eTable 5. **Maternal and child outcome comparison among postnatal depression dyads and SSRI-treated non-postnatal depression dyads **eTable 6. **Associations between postnatal maternal depression, SSRI treatment and the study outcomes in sample without postnatal depression diagnosis **eFigure. **The study population

Supplement 2.
Data Sharing Statement
