# Supplementary material for: Long-Term Maternal and Child Outcomes Following Postnatal SSRI Treatment
Source: JAMA Netw Open. 2023 Aug 29;6(8):e2331270. doi: 10.1001/jamanetworkopen.2023.31270 (PMC10466165; doi:10.1001/jamanetworkopen.2023.31270)
Supplement: Supplement 1. — eMethods. eTable 1. Maternal and child outcomes and the association between postnatal maternal depression, SSRI treatment and the study outcomes in the whole study population eTable 2. Maternal and child outcomes and the association between postnatal maternal depression, SSRI treatment and the study outcomes among PND dyads eTable 3. Association between postnatal maternal depression, SSRI treatment and the study outcomes in the whole study sample controlling for prenatal SSRI use eTable 4. Association between postnatal maternal depression, SSRI treatment and the study outcomes in the postnatal depression sample controlling for prenatal SSRI use eTable 5. Maternal and child outcome comparison among postnatal depression dyads and SSRI-treated non-postnatal depression dyads eTable 6. Associations between postnatal maternal depression, SSRI treatment and the study outcomes in sample without postnatal depression diagnosis eFigure. The study population [file jamanetwopen-e2331270-s001.pdf]

Supplemental Online Content

Liu C-Y, Ystrom E, McAdmas TA. Long-term maternal and child outcomes following postnatal SSRI treatment. *JAMA Netw Open*. 2023;6(8):e2331270.
doi:10.1001/jamanetworkopen.2023.31270

eMethods.
eTable 1. Maternal and child outcomes and the association between postnatal maternal depression, SSRI treatment and the study outcomes in the whole study population
eTable 2. Maternal and child outcomes and the association between postnatal maternal depression, SSRI treatment and the study outcomes among PND dyads
eTable 3. Association between postnatal maternal depression, SSRI treatment and the study outcomes in the **w**hole study sample controlling for prenatal SSRI use
eTable 4. Association between postnatal maternal depression, SSRI treatment and the study outcomes in the postnatal depression sample controlling for prenatal SSRI use
eTable 5. Maternal and child outcome comparison among postnatal depression dyads and SSRI-treated non-postnatal depression dyads
eTable 6. Associations between postnatal maternal depression, SSRI treatment and the study outcomes in sample without postnatal depression diagnosis
eFigure. The study population

This supplemental material has been provided by the authors to give readers additional information about their work.

d

© 2023 Liu C-Y et al. *JAMA Network Open.*

eMethod

The current study used propensity score adjustment method to control for confounding in observational data. This is because given the low numbers of people in our sample who could be categorized as being postnatally depressed and treated with SSRI, running an emulated trial focused only on the extreme end of the depression distribution resulted in the loss of an enormous amount of (in our opinion very useful) data, and a huge loss of statistical power. To retain more study participants for statistical efficiency and to control for potential confounding, we chose to use the propensity scores covariate adjustment approach. In addition, depression and all of the outcomes we focused on are continuously distributed throughout the population, and we have continuous measures on all of these variables (including depression symptoms) for every participant. We therefore chose to use the entire sample to estimate associations between postnatal depressive symptoms and our outcomes, and to ask whether SSRI use for postnatal depression moderates any of these associations. The propensity score was used to adjust for prenatal confounding factors that may have influenced the probability of postnatal SSRI treatment assignment. Previous studies have shown that propensity scores covariate adjustment can provide robust estimates in similar study designs (Elze et al., 2017; Nezvalova-Henriksen et al., 2016). In addition, we used the 'doubly robust' method that incorporates potential confounding, the propensity score and covariates in the final regression to better adjust for potential confounding paths and to compensate for potential misspecification of the propensity score model (Funk et al., 2011). Indeed, we found that including prenatal confounding and the propensity score in the regression improved model performance. To examine robustness of our findings, we performed analyses using propensity score matching method (findings available upon request). In propensity score matching model, associations between postnatal depression and most of our study outcomes became non-significant and non-precise with very wide confidence interval due to the very small sample size (n=177 in non-treated and treated group). Nevertheless, we still found that postnatal SSRI treatment significantly mitigated the negative association between postnatal depression and maternal relationship satisfaction at postpartum month 6.

© 2023 Liu C-Y et al. *JAMA Network Open.*

Elze, M. C., Gregson, J., Baber, U., Williamson, E., Sartori, S., Mehran, R., Nichols, M., Stone, G. W., & Pocock, S. J. (2017). Comparison of Propensity Score Methods and Covariate Adjustment: Evaluation in 4 Cardiovascular Studies. *J Am Coll Cardiol*, *69*(3), 345-357. https://doi.org/10.1016/j.jacc.2016.10.060

Funk, M. J., Westreich, D., Wiesen, C., Sturmer, T., Brookhart, M. A., & Davidian, M. (2011). Doubly robust estimation of causal effects. *Am J Epidemiol*, *173*(7), 761-767. https://doi.org/10.1093/aje/kwq439

Nezvalova-Henriksen, K., Spigset, O., Brandlistuen, R. E., Ystrom, E., Koren, G., & Nordeng, H. (2016). Effect of prenatal selective serotonin reuptake inhibitor (SSRI) exposure on birthweight and gestational age: a sibling-controlled cohort study. *Int J Epidemiol*, *45*(6), 2018-2029. https://doi.org/10.1093/ije/dyw049

© 2023 Liu C-Y et al. *JAMA Network Open.*

eTable 1 Maternal and child outcomes and the association between postnatal maternal depression, SSRI treatment and the study outcomes in the whole study population

			Multivariable model with interaction			
	Non-PND n= 52410 mean (sd)	PND N=8671 mean (sd)	PND Adjusted ß (CI)	P value	PND*SSRI ß (CI)	P value
Maternal outcomes						
SCL-8, mean (SD)						
Postpartum month 6	9.20 (1.69)	13.80 (4.14)	-		-	
postpartum year 1.5	9.73 (2.32)	13.09 (4.24)	0.46 (0.45, 0.46)	<0.001	-0.08 (-0.12, -0.04)	<0.001
postpartum year 3	9.74 (2.56)	12.84 (4.46)	0.38 (0.37, 0.39)	<0.001	-0.02 (-0.07, 0.03)	0.366

© 2023 Liu C-Y et al. *JAMA Network Open.*

postpartum year 5	9.35 (2.25)	11.65 (4.01)	0.33 (0.31, 0.34)	<0.001	-0.08 (-0.14, -0.02)	0.014
Relationship satisfaction						
postpartum month 6	53.81 (6.06)	47.85 (9.29)	-0.31 (-0.32, -0.30)	<0.001	0.17 (0.13, 0.21)	<0.001
postpartum year 1.5	52.76 (7.04)	47.75 (9.44)	-0.22 (-0.23, -0.21)	<0.001	0.14 (0.09, 0.19)	<0.001
postpartum year 3	51.13 (8.28)	46.17 (10.39)	-0.19 (-0.20, -0.18)	<0.001	0.14 (0.08, 0.20)	<0.001
Child outcomes						
Internalizing problems						
1.5y	3.84 (0.88)	4.07 (0.99)	0.09 (0.07, 0.10)	<0.001	-0.04 (-0.09, 0.01)	0.253
3 y	3.68 (0.89)	3.95 (1.07)	0.10 (0.09, 0.12)	<0.001	-0.02 (-0.08, 0.04)	0.653
5 y	3.51 (0.84)	3.77 (1.05)	0.10 (0.08, 0.11)	<0.001	-0.06 (-0.13, 0.02)	0.253

© 2023 Liu C-Y et al. *JAMA Network Open.*

Externalizing problems						
1.5y	11.81 (2.19)	12.49 (2.39)	0.11 (0.10, 0.12)	<0.001	-0.08 (-0.14, -0.03)	0.006
3 y	11.74 (2.36)	12.44 (2.57)	0.12 (0.11, 0.13)	<0.001	-0.05 (-0.10, 0.01)	0.253
5 y	10.37 (2.18)	11.08 (2.58)	0.13 (0.12, 0.15)	<0.001	-0.13 (-0.21, -0.06)	0.002
ADHD 5y	16.16 (4.33)	17.75 (5.61)	0.14 (0.13, 0.16)	<0.001	-0.17 (-0.24, -0.10)	<0.001
Motor development						
1.5y	23.05 (1.54)	22.87 (1.86)	-0.05 (-0.06, -0.04)	<0.001	-0.02 (-0.07, 0.03)	0.653
3y	21.67 (2.67)	21.56 (2.74)	-0.05 (-0.06, -0.04)	<0.001	0.00 (-0.06, 0.06)	0.911
Language development						
1.5y	13.52 (1.74)	13.33 (1.92)	-0.05 (-0.06, -0.04)	<0.001	0.01 (-0.05, 0.06)	0.911
3y	14.48 (0.92)	14.33 (1.16)	-0.06 (-0.07, -0.04)	<0.001	0.02 (-0.04, 0.08)	0.653

a. PND: postnatal depression. The model examined associations between postnatal maternal depression measured by the SCL-8 at postpartum month 6 and study outcomes

© 2023 Liu C-Y et al. *JAMA Network Open.*

b. Adjusted ß, standardised regression coefficient adjusted for covariates
c. Covariate for maternal outcomes include maternal age, parity, maternal income, prenatal depression & anxiety, lifetime depression, income, education, propensity score, and maternal prenatal alcohol and tobacco use.
d. Covariates for child outcomes include maternal age, parity, maternal income, prenatal depression & anxiety, lifetime depression, income, education, propensity score, maternal prenatal alcohol and tobacco use, child sex, birthweight, gestational age.
e. PND *SSRI: moderation terms in the interaction model

© 2023 Liu C-Y et al. *JAMA Network Open.*

eTable 2 Maternal and child outcomes and the association between postnatal maternal depression, SSRI treatment and the study outcomes among PND dyads

			Multivariable model with interaction			
	Non-SSRI-treated PND n=8494 mean (sd)	SSRI-Treated PND n=177 mean (sd)	PND Adjusted ß (CI)	P value	PND*SSRI ß (CI)	P value
Maternal outcomes						
SCL-8, mean (SD)						
Postpartum month 6	13.72 (4.07)	17.70 (5.25)	-		-	
postpartum year 1.5	13.04 (4.19)	15.37 (5.48)	0.36 (0.34, 0.38) ***	<0.001	-0.06 (-0.13, 0.00)	0.102
postpartum year 3	12.79 (4.43)	15.62 (5.06)	0.30 (0.27, 0.32) ***	<0.001	0.02 (-0.08, 0.08)	0.996

© 2023 Liu C-Y et al. *JAMA Network Open.*

postpartum year 5	11.61 (3.97)	13.34 (5.52)	0.27 (0.24, 0.31) ***	<0.001	-0.06 (-0.16, 0.04)	0.264
Relationship satisfaction						
postpartum month 6	47.83 (9.29)	49.24 (9.73)	-0.17 (-0.20, -0.15) ***	<0.001	0.13 (0.07, 0.19)	<0.001
postpartum year 1.5	47.74 (9.42)	49.03 (10.10)	-0.10 (-0.13, -0.08) ***	<0.001	0.11 (0.05, 0.18)	0.003
postpartum year 3	46.16(10.38)	46.75 (10.97)	-0.09 (-0.12, -0.06) ***	<0.001	0.12 (0.04, 0.19)	0.003
Child outcomes						
Internalizing problems						
1.5y	4.07 (0.99)	4.11 (1.08)	0.05 (0.03, 0.07) ***	<0.001	-0.02 (-0.08, 0.04)	0.791
3 y	3.95 (1.07)	4.01 (1.10)	0.05 (0.03, 0.08) ***	<0.001	0.01 (-0.06, 0.08)	0.965
5 y	3.77 (1.05)	3.96 (1.22)	0.06 (0.03, 0.09) ***	<0.001	-0.04 (-0.13, 0.05)	0.778

© 2023 Liu C-Y et al. *JAMA Network Open.*

Externalizing problems						
1.5y	12.50 (2.39)	12.17 (2.33)	0.04 (0.02, 0.06)	<0.001	-0.05 (-0.11, 0.01)	0.334
3y	12.44 (2.57)	12.54 (2.80)	0.05 (0.02, 0.07)	<0.001	0.00 (-0.06, 0.07)	0.993
5y	11.08 (2.58)	10.82 (2.04)	0.07 (0.04, 0.10)	<0.001	-0.11 (-0.19, 0.02)	0.089
ADHD	17.76 (5.64)	17.59 (4.47)	0.08 (0.04, 0.11)	<0.001	-0.15 (-0.24, -0.05)	0.021
5y						
Motor development						
1.5y	22.88 (1.86)	22.56 (1.93)	-0.03 (-0.06, 0.01)	0.01	-0.03 (-0.10, 0.03)	0.778
3y	21.56 (2.74)	21.49 (2.62)	-0.03 (-0.06, -0.01)	0.01	-0.03 (-0.09, 0.04)	0.778
Language development						
1.5y	13.33 (1.16)	13.03 (2.00)	-0.03 (-0.06, -0.01)	0.01	-0.01 (-0.07, 0.05)	0.965
3y	14.33 (1.92)	14.30 (1.09)	-0.01 (-0.04, 0.02)	0.55	-0.01 (-0.08, 0.07)	0.965

© 2023 Liu C-Y et al. *JAMA Network Open.*

a. PND: postnatal depression. The model examined associations between postnatal maternal depression measured by the SCL-8 at postpartum month 6 and study outcomes
b. Adjusted ß, standardised regression coefficient adjusted for covariates
c. Covariate for maternal outcomes include maternal age, parity, maternal income, prenatal depression, lifetime depression, income, education, propensity score, and maternal prenatal alcohol and tobacco use.
d. Covariates for child outcomes include maternal age, parity, maternal income, prenatal depression, lifetime depression, income, education, propensity score, maternal prenatal alcohol and tobacco use, child sex, birthweight, gestational age.
e. PND*SSRI: moderation terms in the interaction model

© 2023 Liu C-Y et al. *JAMA Network Open.*

eTable 3 Association between postnatal maternal depression, SSRI treatment and the study outcomes in the **whole study sample** controlling for prenatal SSRI use

	Multivariable model with interaction	
	PND<br>Adjusted ß (CI)	PND*SSRI<br>ß (CI)
Maternal outcomes		
SCL-8, mean (SD)<br>year 1.5	0.46 (0.45, 0.46) ***	-0.08 (-0.12, -0.04) ***
year 3	0.38 (0.37, 0.39) ***	-0.02 (-0.07, 0.03)
year 5	0.33 (0.31, 0.34) ***	-0.08 (-0.15, -0.02) *
Relationship satisfaction		
month 6	-0.31 (-0.32, -0.30) ***	0.16 (0.12, 0.20) ***
year 1.5	-0.22 (-0.23, -0.21) ***	0.14 (0.09, 0.19) ***
year 3	-0.19 (-0.20, -0.18) ***	0.14 (0.08, 0.20) ***
Child outcomes		

© 2023 Liu C-Y et al. *JAMA Network Open.*

Internalizing problems		
1.5y	0.09 (0.07, 0.10) ***	-0.04 (-0.09, 0.01)
3 y	0.10 (0.09, 0.12) ***	-0.02 (-0.08, 0.03)
5 y	0.10 (0.08, 0.11) ***	-0.06 (-0.13, 0.01)
Externalizing problems		
1.5y	0.11 (0.10, 0.12) ***	-0.08 (-0.13, -0.03) *
3 y	0.12 (-0.11, 0.13) ***	-0.04 (-0.10, 0.01)
5 y	0.13 (0.12, 0.15) ***	-0.13 (-0.20, -0.05) ***
ADHD 5y	0.14 (0.13, 0.16) ***	-0.16 (-0.24, -0.09) ***
Motor development		
1.5y	-0.05 (-0.06, -0.04) ***	-0.02 (-0.07, 0.04)
3y	-0.05 (-0.06, -0.04) ***	0.00 (-0.06, 0.06)
Language development		
1.5y	-0.05 (-0.06, -0.04) ***	0.01 (-0.05, 0.06)
3y	-0.06 (-0.07, -0.04) ***	0.01(-0.05, 0.07)

a. A total of 1051 children in the whole study sample were exposed to SSRI during pregnancy (619 in non-postnatal depression control and 432 in postnatal depression groups)

© 2023 Liu C-Y et al. *JAMA Network Open.*

eTable 4 Association between postnatal maternal depression, SSRI treatment and the study outcomes in the **Postnatal depression sample** controlling for prenatal SSRI use

	Multivariable model with interaction	
	PND<br>Adjusted ß (CI)	PND*SSRI<br>ß (CI)
Maternal outcomes		
SCL-8, mean (SD)<br>year 1.5	0.36 (0.34, 0.38) ***	-0.06 (-0.13, 0.00)
year 3	0.30 (0.27, 0.32) ***	0.00 (-0.08, 0.08)
year 5	0.27 (0.24, 0.31) ***	-0.06 (-0.16, 0.04)
Relationship satisfaction		
month 6<br>year 1.5<br>year 3	-0.17 (-0.20, -0.15) ***<br>-0.10 (-0.13, -0.08) ***<br>-0.09 (-0.12, -0.06) ***	0.12 (0.06, 0.18) ***<br>0.11 (0.04, 0.17) ***<br>0.11 (0.04, 0.19) ***
Child outcomes		

© 2023 Liu C-Y et al. *JAMA Network Open.*

Internalizing problems		
1.5y	0.05 (0.03, 0.07) ***	-0.02 (-0.08, 0.04)
3 y	0.05 (0.03, 0.08) ***	0.01 (-0.06, 0.08)
5 y	0.06 (0.03, 0.09) ***	-0.03 (-0.12, 0.06)
Externalizing problems		
1.5y	0.04 (0.02, 0.06) ***	-0.05 (-0.10, 0.01)
3 y	0.05 (0.02, 0.07) ***	0.00 (-0.06, 0.07)
5 y	0.07 (0.04, 0.10) ***	-0.08 (-0.17, 0.00)
ADHD 5y	0.08 (0.04, 0.11) ***	-0.13 (-0.22, -0.04)
Motor development		
1.5y	-0.03 (-0.06, -0.01) *	-0.03 (-0.10, 0.03)
3y	-0.03 (-0.06, -0.01) *	-0.02 (-0.08, 0.04)
Language development		
1.5y	-0.03 (-0.06, -0.01) ***	0.00 (-0.06, 0.06)
3y	-0.01 (-0.04, 0.02)	-0.01 (-0.09, 0.06)

a. A total of 352 children in the non-SSRI treated PND dyads were exposed to prenatal SSRI, 80 children in the SSRI treated PND dyads were exposed to prenatal SSRI

© 2023 Liu C-Y et al. *JAMA Network Open.*

eTable 5 Maternal and child outcome comparison among postnatal depression dyads and SSRI-treated non-postnatal depression dyads

	Non-treated PND dyads [a] n=8494 mean (sd)	Treated PND dyads n=177 [b] mean (sd)	SSRI treated non-PND dyads [c] n=179 mean (sd)	Effect size	Post-hoc comparison
Maternal outcomes					
Maternal mental health (SCL-8)					
postpartum month 6	13.72 (4.07)	17.70 (5.25)	10.88 (2.44)	0.03	b>a>c
postpartum year 1.5	13.04 (4.19)	15.37 (5.48)	12.08 (3.70)	0.004	b>a>c
postpartum year 3	12.79 (4.43)	15.62 (5.06)	12.51 (4.51)	0.004	b>a, b>c
postpartum year 5	11.61 (3.97)	13.34 (5.52)	11.12 (3.79)	0	b>a, b>c
Maternal report of relationship satisfaction					
postpartum month 6	47.83 (9.29)	49.24 (9.73)	52.60 (6.47)	0.006	c>a>b

© 2023 Liu C-Y et al. *JAMA Network Open.*

postpartum year 1.5	47.74 (9.42)	49.03 (10.10)	49.42 (9.15)	0.001	c>a
postpartum year 3	46.16(10.38)	46.75 (10.97)	46.65 (9.33)	-	-
Child outcomes					
Internalizing problems					
1.5y	4.07 (0.99)	4.11 (1.08)	3.93 (0.93)	-	-
3 y	3.95 (1.07)	4.01 (1.10)	3.88 (1.01)	-	-
5 y	3.77 (1.05)	3.96 (1.22)	3.84 (1.21)	-	-
Externalizing problems					
1.5y	12.50 (2.39)	12.17 (2.33)	11.91 (2.32)	0.001	a>c
3 y	12.44 (2.57)	12.54 (2.80)	12.11 (2.63)	-	-
5 y	11.08 (2.58)	10.82 (2.04)	10.85 (2.33)	-	-
ADHD 5y	17.76 (5.64)	17.59 (4.47)	17.57 (6.5)	-	-
Motor development					
1.5y	22.88 (1.86)	22.56 (1.93)	22.41 (2.33)	0.001	a>b
3y	21.56 (2.74)	21.49 (2.62)	21.10 (2.94)	-	-
Language development					

© 2023 Liu C-Y et al. *JAMA Network Open.*

1.5y	13.33 (1.16)	13.03 (2.00)	12.79 (2.09)	0.002	a>c
3y	14.33 (1.92)	14.30 (1.09)	14.22 (1.39)	-	-

a. Kruskal-Wallis Test was used for outcome comparison; Dunn test was used for post-hoc analysis
b. Epsilon-squared was used for effect size statistics for Kruskal-Wallis Test: small effect <0.08, medium effect 0.08- <0.26, large effect ≥0.26

© 2023 Liu C-Y et al. *JAMA Network Open.*

eTable 6 Associations between postnatal maternal depression, SSRI treatment and the study outcomes in sample without postnatal depression diagnosis

			Multivariable model with interaction	
	Non-PND control n=52410 mean (sd)	SSRI-Treated non-PND n=179 mean (sd)	PND Adjusted ß (CI)	PND *SSRI ß (CI)
Maternal age	30.41 (4.31)	31.20 (4.48)	-	-
Parity	0.74 (0.85)	0.79 (0.92)	-	-
Education	4.74 (1.16)	4.43 (1.35)	-	-
Income	4.23 (1.32)	3.89 (1.34)	-	-
Prenatal alcohol use (N, %)	14417 (27.6)	47 (26.3)	-	-
Prenatal tobacco use (N, %)	2506 (4.8)	14 (7.8)	-	-
Prenatal SCL-8	9.58 (2.13)	12.28 (4.50)	-	-

© 2023 Liu C-Y et al. *JAMA Network Open.*

Lifetime depression (N, %)	2247 (4.3)	108 (60.3)		
Maternal outcomes				
EPDS, mean (SD) Postpartum month 6	1.98 (1.93)	3.21 (2.04)	-	-
SCL-8, mean (SD)				
Month 6	9.20 (1.68)	10.88 (2.44)	-	-
year 1.5	9.73 (2.32)	12.08 (3.70)	0.47 (0.46, 0.49) ***	-0.04 (-0.18, 0.11)
year 3	9.74 (2.56)	12.51 (4.51)	0.40 (0.38, 0.41) ***	0.14 (-0.03, 0.30)
year 5	9.35 (2.25)	11.12 (3.79)	0.35 (0.34, 0.37) ***	-0.35 (-0.58, -0.13) **
Relationship satisfaction				
month 6	53.81 (6.04)	52.60 (6.47)	-0.31 (-0.32, -0.30) ***	0.04 (-0.11, 0.17)
year 1.5	52.76 (7.01)	49.42 (9.15)	-0.25 (-0.27, -0.24) ***	0.09 (-0.11, 0.26)
year 3	51.10 (8.26)	46.65 (9.33)	-0.22 (-0.24, -0.20) ***	-0.12 (-0.36, 0.07)

© 2023 Liu C-Y et al. *JAMA Network Open.*

Child outcomes				
Sex (male) (N, %)	26732 (51)	98 (54.7)		
Birthweight (kg)	3.59 (0.55)	3.51 (0.56)		
Gestational age (wks)	39.74 (2.32)	39.37 (2.09)		
Internalizing problems				
1.5y	3.84 (0.88)	3.93 (0.93)	0.11 (0.09, 0.13) ***	0.17 (-0.04, 0.39)
3 y	3.68 (0.89)	3.88 (1.01)	0.14 (0.12, 0.16) ***	-0.04 (-0.27, 0.20)
5 y	3.51 (0.84)	3.84 (1.21)	0.12 (0.09, 0.14) ***	0.02 (-0.25, 0.29)
Externalizing problems				
1.5y	11.81 (2.19)	11.91 (2.32)	0.17 (0.15, 0.18) ***	-0.13 (-0.32, 0.12)
3 y	11.74 (2.36)	12.11 (2.63)	0.18 (0.16, 0.20) ***	-0.02 (-0.23, 0.25)
5 y	10.37 (2.18)	10.85 (2.33)	0.18 (0.16, 0.20) ***	-0.13 (-0.37, 0.16)
ADHD 5y	16.16 (4.33)	17.57 (6.5)	0.19 (0.17, 0.21) ***	-0.04 (-0.30,0.22)
Motor development				
1.5y	23.05 (1.54)	22.41 (2.33)	-0.07 (-0.09, -0.05) ***	-0.24 (-0.47, -0.02)
3y	21.67 (2.67)	21.10 (2.94)	-0.08 (-0.10, -0.06) ***	-0.08 (-0.32, 0.16)

© 2023 Liu C-Y et al. *JAMA Network Open.*

Language development				
1.5y	13.52 (1.74)	12.79 (2.09)	-0.07 (-0.09, -0.05) ***	-0.11 (-0.33, 0.12)
3y	14.48 (0.92)	14.22 (1.39)	-0.09 (-0.11, -0.07) ***	-0.30 (-0.53, -0.08)

a. PND: postnatal depression. The model examined associations between postnatal maternal depression measured by the SCL-8 at postpartum month 6 and study outcomes
b. Adjusted ß, standardised regression coefficient adjusted for covariates
c. Covariate for maternal outcomes include maternal age, parity, maternal income, prenatal depression & anxiety, lifetime depression, income, education, propensity score, and maternal prenatal alcohol and tobacco use.
d. Covariates for child outcomes include maternal age, parity, maternal income, prenatal depression & anxiety, lifetime depression, income, education, propensity score, maternal prenatal alcohol and tobacco use, child sex, birthweight, gestational age.
e. PND*SSRI: moderation terms in the interaction model
f. Reported p value were adjusted for multiple comparison (FDR 5%), ***p<0.001, ** p<0.01, *p<0.05

© 2023 Liu C-Y et al. *JAMA Network Open.*

eFigure 1 The study population

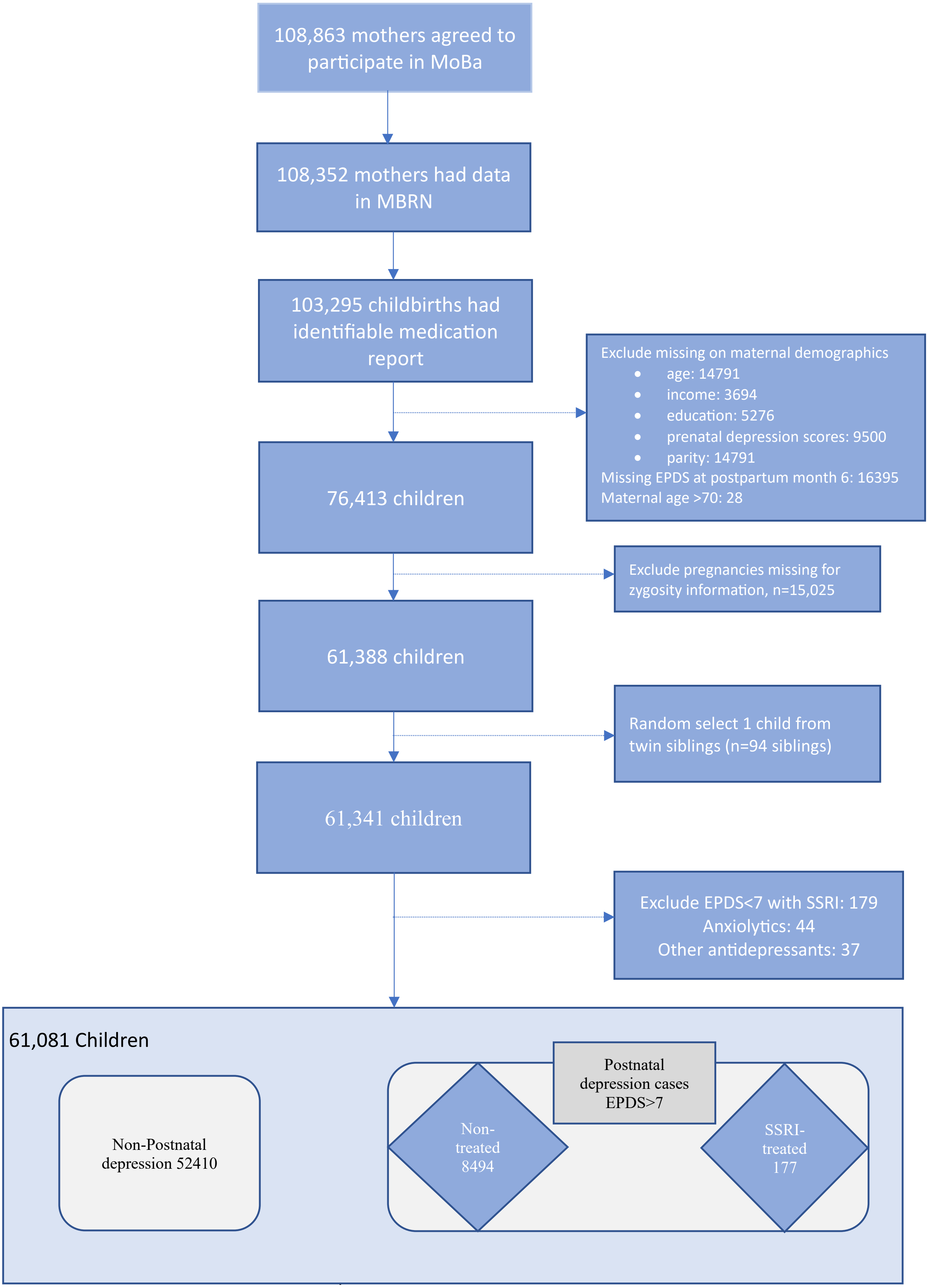

© 2023 Liu C-Y et al. *JAMA Network Open.*
