# Supplementary material for: Long-Term Maternal and Child Outcomes Following Postnatal SSRI Treatment
Source: JAMA Netw Open. 2023 Aug 29;6(8):e2331270. doi: 10.1001/jamanetworkopen.2023.31270 (PMC10466165; doi:10.1001/jamanetworkopen.2023.31270)
Supplement: Supplement 2. — Data Sharing Statement [file jamanetwopen-e2331270-s002.pdf]

Data Sharing Statement

Liu. Long-Term Maternal and Child Outcomes Following Postnatal SSRI Treatment. *JAMA Netw Open*. Published August 29, 2023. doi:10.1001/jamanetworkopen.2023.31270

Data
Data available: Yes
Data types: Data dictionary
How to access data: Data dictionary can be accessed on the MoBa cohort website. https://www.fhi.no/en/studies/moba/for-forskere-artikler/questionnaires-from-moba/
When available: With publication

Supporting Documents
Document types: Statistical/analytic code
How to access documents: Analytic code can be provided upon request. Please send email to the corresponding author at Kate.liu@kcl.ac.uk
When available: With publication

Additional Information
Who can access the data: researchers whose proposed use of the data has been approved
Types of analyses: For a specified purpose
Mechanisms of data availability: after approval of a proposal
